# The Issue as a Counter-irritant

**Published:** 1879-01

**Authors:** P. Harold Hayes


### THE ISSUE AS A MODE OF COUNTER-IRRITATION.

BY P. HAROLD HAYES, M. D.

Doubtless the most ancient—certainly the most universal of all remedial procedures—is Counter-irritation. Prof. Thomson says he found it "to constitute two-thirds of all the therapeutics of the Bedouin Arabs, at the present day, and he further says Birmingham supplies the great Chinese Empire with needles, but not for sewing clothes, they are for pricking patients. A single celestial colleague of ours will use one to three papers of needles on a single hapless victim of malarial fever." The *issue*, as a means of counter-irritation and evacuation, is mentioned in the oldest records of medicine. Nature herself taught the observing physician that a sore was the outlet for vile humors, and that thus internal and vital organs were relieved. In the present age, characterized as it is by diseases of the nervous system, rather than those of the vascular and glandular systems, the issue has fallen into great disuse. Physicians, generally, so far from being aware of their occasional great value, are even generally ignorant of how to make them, and of what indications they may be expected to fulfil. Once having had his attention directed to this matter, once observation is on the *qui vive* as to this utility, the physician will be surprised by the number of illustrations nature or accident, have prepared for us of the virtues of an issue. One of the most interesting cases of this kind I can now recall is that of Capt. Speke, the traveler, who, while carrying on his explorations in the heart of Africa, suffered terribly from inflammation of the eyes, so common in that country. Just at this time he was stung by a poisonous insect in one ear, and a free, purulent discharge immediately followed the sting, and this cured his eyes.

An elderly woman living in an adjoining town, who had suffered from asthma for many years, was thrown from her carriage and received a compound fracture of the leg. The discharge and the irritation at the point of the fracture perfectly cured her asthma. A woman in middle life came to me, with an old, irritable and very painful ulcer just above her left ankle, which she wished me to cure. It came out, however, in her history that she had formerly suffered very severely from the asthma, and from facial neuralgia; but that since the ulcer appeared she had not suffered, in the least, from either of these diseases.

I have now used the issue in my practice in chronic diseases for several years, and I have found it to be a remedy of great power in properly chosen cases. Using it at first in a case of sciatica successfully, I went on trying it until I discovered its untold value in a great variety of chronic diseases. I now take it for granted, alike with physician and patient, what is capable of the most abundant proof and illustration, from the days of Hippocrates to those of Von Ziemssen, that there are in nearly all chronic diseases morbific humors in the blood, and that these settling upon some organ of the body are at the bottom of our diseases. It thus comes to pass in so many instances that evacuation is a first principle of cure. The issue acts by counter-irritation and by evacuation; it is the discharge mainly that cures, and it takes off the burden from affected organs. And now, nature being newly disposed to a cure, everything else done for the case does double the good it otherwise could have done. Moreover, vigorous local treatment may now be used, without any danger of driving disease from one organ or part to another as from the womb to the brain, lungs, stomach, or spine, or a skin disease to the lungs, womb, spine, or bowels. The issue is a sort of pathological divining cup. For you can judge by its aspects and behavior, its color, quality of the granulations, appearances of the discharges, rapidity of exfoliation of the slough, as to the quality of your patient's blood, the intensity of any dyscrasia you may have discovered, and thus it will be a sort of index to your patient's condition and progress under treatment. The disease thus shows itself in and tends to spend itself on the issue. And now is the time for the physician thus aided to use all forms of constitutional medicines and hygienic agencies to change the habit of disease into the established habit of health.

I will add to what I have said a few cases from my note-book:

M.—A man in middle life was a prisoner at Andersonville during the war. He has had dyspepsia ever since, and lately a terrible gastrodynia coming on as the stomach begins to get empty, forenoon and afternoon. The issue not only cured the gastrodynia, but the dyspepsia and constipation as well.

G.—*Acne* in all stages; face inflamed; skin thickened and tuberculated. He says the issue in his arm takes away *heat* and *irritation* from his face. He kept his issue open more than a year. He would not be without it. Both local and constitutional treatment worked like a charm with the help of the issue, and this young man, who had previously spent ten years in metropolitan cities under treatment, was cured in a year's time, and came out at the end ruddy, fat and vigorous.

D.—A rugged farmer of fifty, contracted deep-seated lumbar pains, nearly a year before, from loading hay in a stooping posture under a scaffold. Had "suffered everything and tried everything" for nearly a year. I put on two issues in the sacro-iliac region. He returned in a month to say the pains were gone, and that he told his wife yesterday that he felt twenty-five years younger than he did a month ago. In the case of a young lady of full habit, who, having had measles in February, began to cough badly in March, I applied the issue to the left arm with the most prompt relief to the harassing night cough. In this manner I drew off heat and irritation from the lungs, and gastric irritation from the stomach. My patient began to digest codliver-oil, hypophosphides and tincture of iron, and she took daily a large amount of out-door exercise with constant benefit to her appetite. Before midsummer she was in perfect health.

There are a great many things to be said as to the making and managing of issues, and the selection of cases, for which I have not time in this article.

